# Supplementary material for: TRABID overexpression enables synthetic lethality to PARP inhibitor via prolonging 53BP1 retention at double-strand breaks
Source: Nat Commun. 2023 Mar 31;14:1810. doi: 10.1038/s41467-023-37499-5 (PMC10066190; doi:10.1038/s41467-023-37499-5)
Supplement: Supplementary file 1 — Supplementary Information [file 41467_2023_37499_MOESM1_ESM.pdf]

Supplementary Information

TRABID overexpression enables synthetic lethality to PARP inhibitor via prolonging 53BP1 retention at double-strand breaks

Jian Ma, Yingke Zhou, Penglin Pan, Haixin Yu, Zixi Wang, Lei Lily Li, Bing Wang, Yuqian Yan, Yunqian Pan, Qi Ye, Tianjie Liu, Xiaoyu Feng, Shan Xu, Ke Wang, Xinyang Wang, Yanlin Jian, Bohan Ma, Yizeng Fan, Yang Gao, Haojie Huang, and Lei Li

This PDF file includes:

Supplementary Figure 1 to 4

Supplementary Figure Legends

References cited in Supplementary Information

Supplementary figure 1

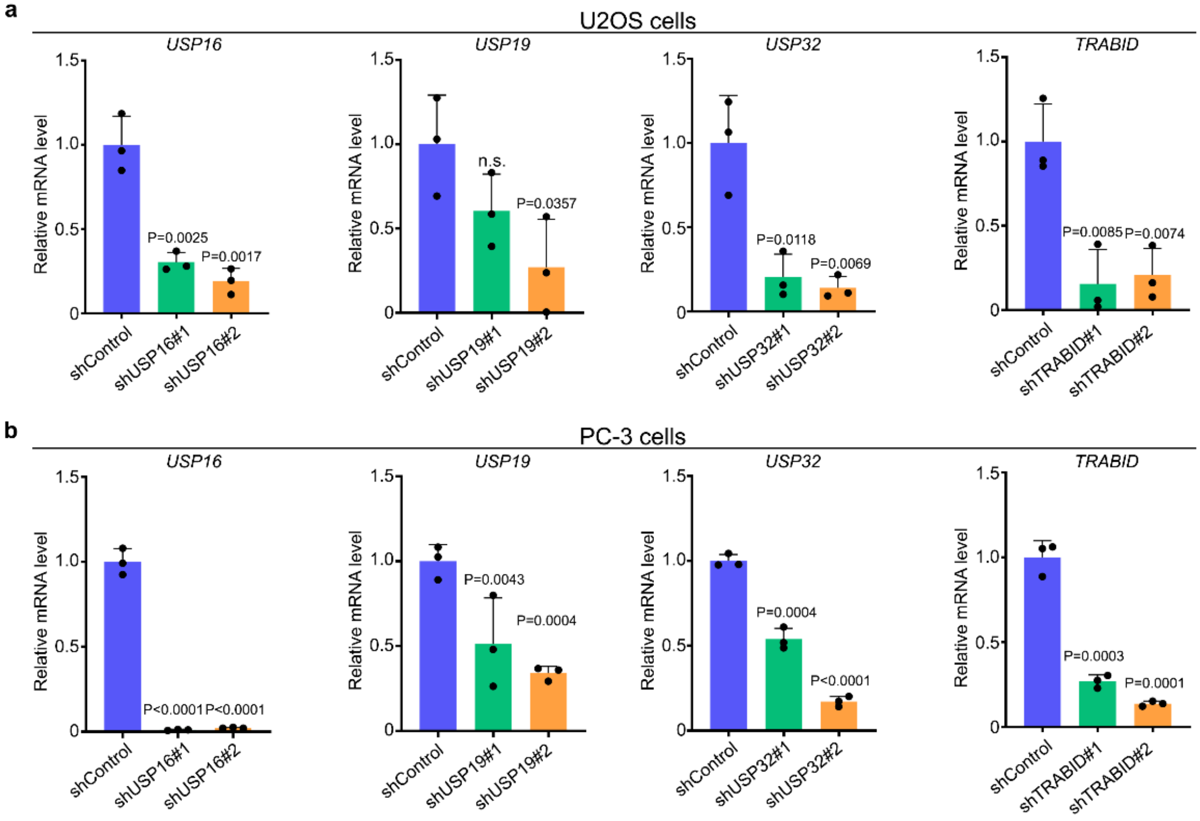

Supplementary Fig. 1 Knockdown efficiencies of deubiquitinase genes, Related to Figure 1

U2OS (**a**) and PC-3 (**b**) cells were infected with lentivirus expressing indicated shRNAs and harvested for RT-qPCR. Data are shown as mean ± SD (n=3). Two-tailed unpaired Student's t-test. P values based on the order of appearance: in **a** (0.0025, 0.0017, 0.1323, 0.0357, 0.0118, 0.0069, 0.0085, 0.0074) and in **b** (2.4E-5, 2.5E-5, 0.0043, 0.0004, 0.0004, 6.8E-6, 0.0003, 0.0001). n.s., not significant. Source data are provided in this paper.

Supplementary figure 2

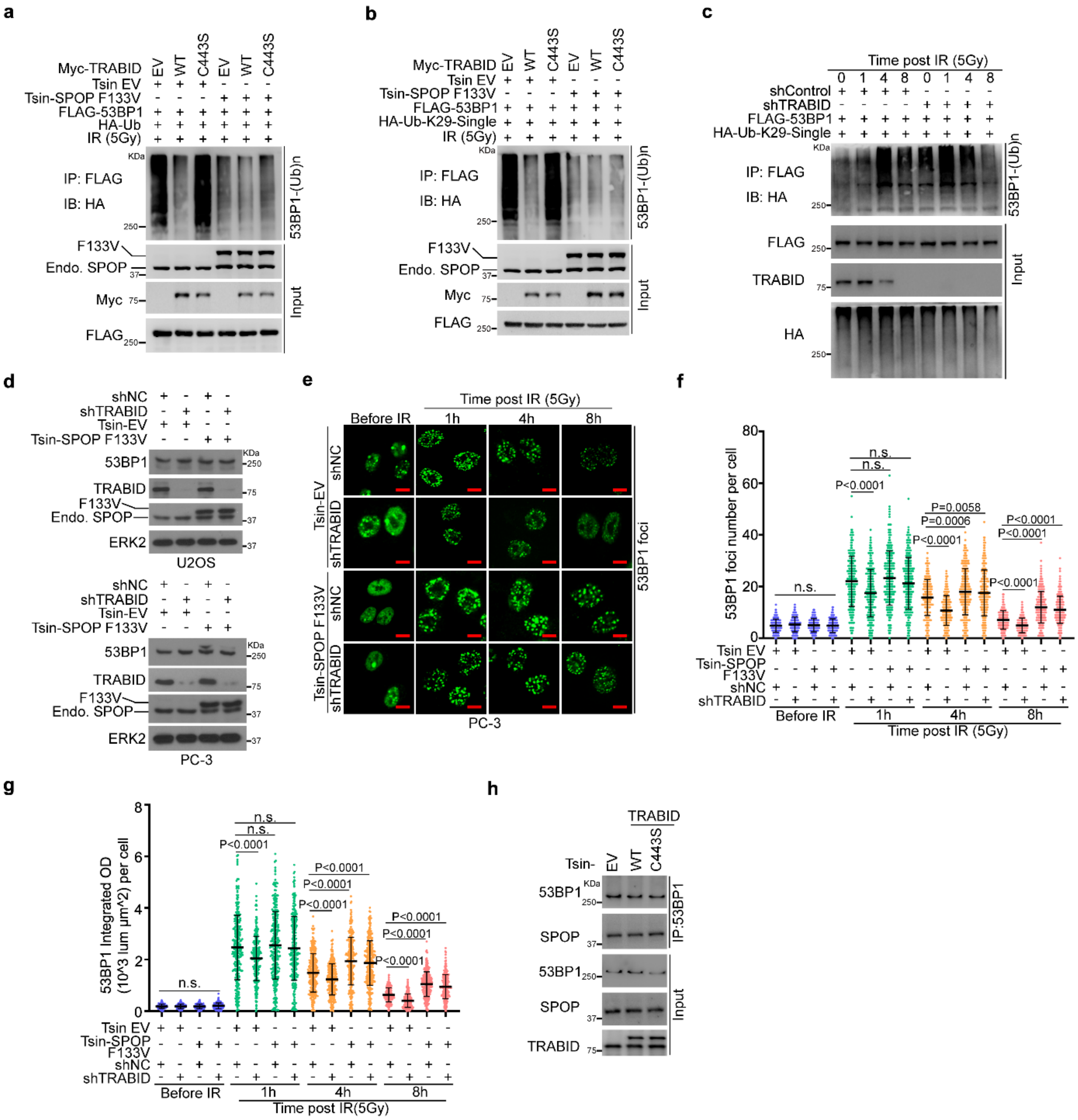

Supplementary Fig. 2 TRABID antagonizes SPOP-mediated removal of 53BP1 from DSB sites, Related to Figure 3

a, b Ubiquitination assay in 293T cells transfected with HA-WT (**a**) or K29-only (**b**) ubiquitin together with other indicated plasmids and treated with IR (5 Gy) for 1 hour.

c Ubiquitination assay in 293T cells transfected with indicated plasmids. Cells were harvested at different time points after IR.

d U2OS (**top**) and PC-3 (**bottom**) cells were infected with lentivirus expressing indicated shRNA and harvested for WB analysis. ERK2 was used as a loading control.

e-g PC-3 cells infected with lentivirus expressing indicated shRNA were treated with IR followed by IFC of 53BP1 at the indicated time points after IR (**e**). Scale bar, 10 μm. The average 53BP1 foci number (**f**) and foci density (**g**) in each cell were quantified. Data are presented as means ± SD of more than 300 cells from three biological replicates. Two-tailed unpaired Student's t-test. n.s., not significant. P values based on the order of appearance: in **f** (0.33, 0.5657, 0.9126, 4.7E-9, 0.1316, 0.3342, 1.6E-20, 0.0006, 0.0058, 4E-15, 5.7E-30, 9.9E-25) and in **g** (0.5466, 0.7991, 0.0932, 1.9E-6, 0.4184, 0.7657, 4.7E-6, 1.1E-10, 1.8E-8, 4.3E-23, 3.3E-34, 3.4E-22).

h PC-3 cells were infected with lentivirus expressing indicated plasmids and harvested for co-IP analysis. Source data are provided in this paper or Mendeley database (https://data.mendeley.com/datasets/n9txt6y5cj/1). Similar results for (**a-d**) and (**h**) panels were obtained in three independent experiments.

Supplementary figure 3

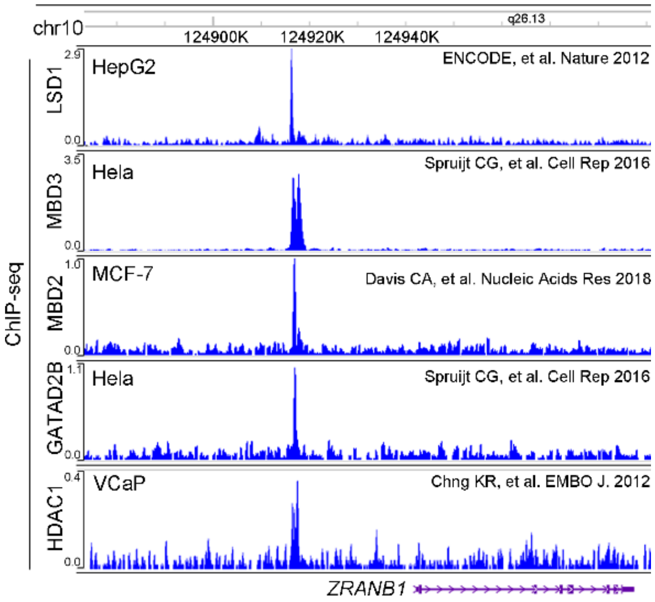

Supplementary Fig. 3 ChIP-seq signal showing the occupancy of the indicated components of the NuRD complex in the ZRANB1 gene locus, Related to Figure 4

Screenshot of the Cistrome Data Browser (http://cistrome.org/db/#/) showing ChIP-seq signal profiles of the indicated components of the NuRD complex (reported previously [1-4]) in the *ZRANB1* gene locus in different human cell lines. The accession codes from Cistrome Data Browser: 101921 (GEO: GSM2825431), 71069 (GEO: GSM2104436), 64901 (ENCODE: ENCSR940MHE_2), 71067 (GEO: GSM2104440), and 8658 (GEO: GSM717399).

Supplementary figure 4

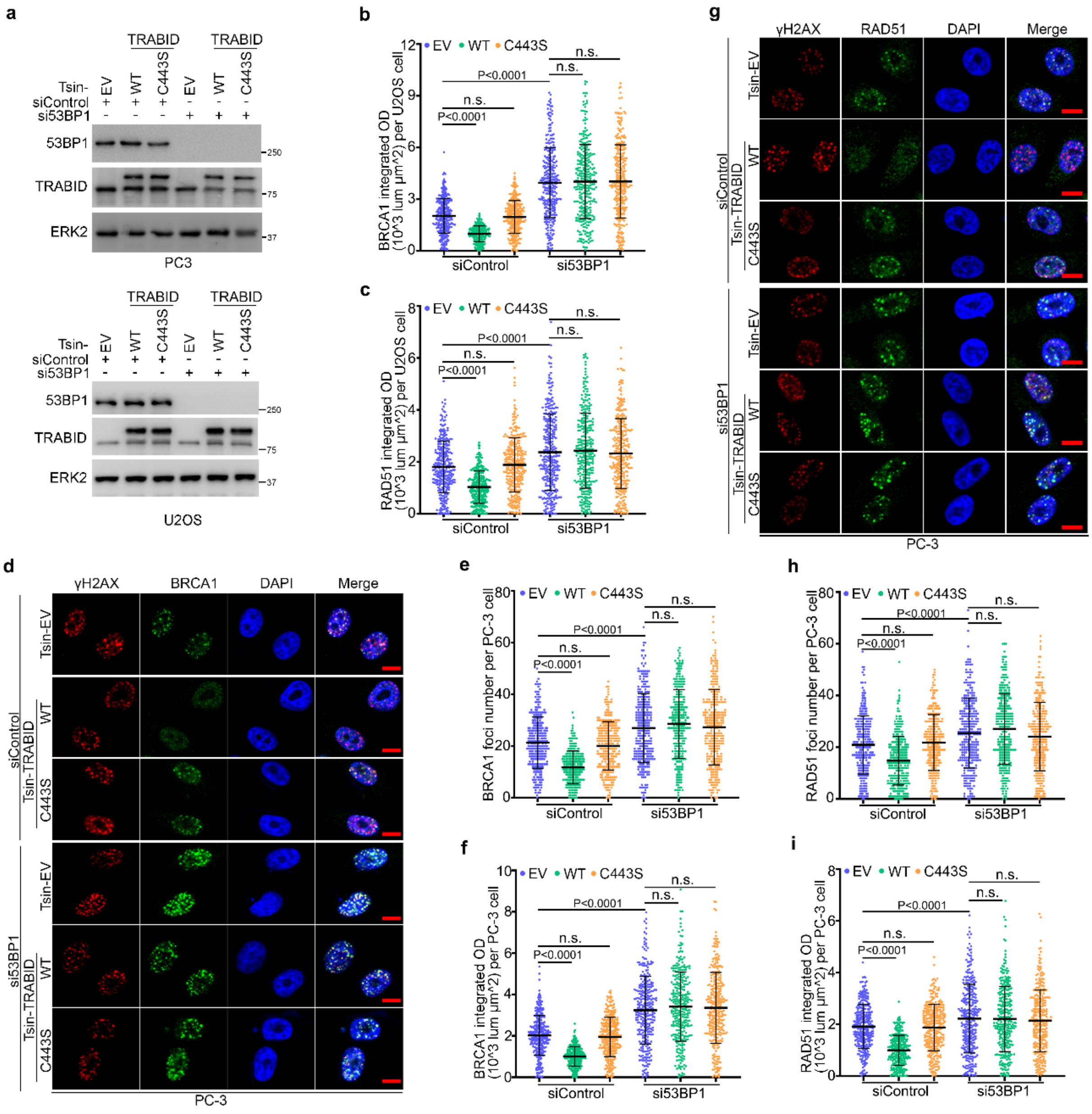

Supplementary Fig. 4 TRABID overexpression inhibits HR activity, Related to Figure 5

a PC-3 (**top**) and U2OS (**bottom**) cells were infected with lentivirus expressing the indicated plasmids and treated with the indicated siRNAs and harvested for WB analysis. ERK2 was used as a loading control. Similar results were obtained in three independent experiments.

b, c U2OS cells infected with lentivirus expressing EV, TRABID-WT or TRABID-C443S and treated with siControl or si53BP1 were exposed to IR. Cells were subjected to IFC of BRCA1 and RAD51 at 1 hour after IR and IFC results are shown in Fig. 5d, f. The average foci density of BRCA1 (**b**) and RAD51 (**c**) in each cell are shown. Data are presented as means ± SD of more than 300 cells from three biological replicates. Two-tailed unpaired Student's t-test. n.s., not significant. P values based on the order of appearance: in **b** (9.6E-50, 0.4198, 4.6E-42, 0.6769, 0.6318) and in **c** (6.5E-28, 0.3313, 4.8E-8, 0.661, 0.6374).

d-i PC-3 cells infected with lentivirus expressing EV, TRABID-WT or TRABID-C443S and treated with siControl or si53BP1 were exposed to IR followed by IFC of BRCA1 (**d**) and RAD51 (**g**) 1 h after IR. Scale bar, 10 μm. The average foci number (**e, h**) and foci density (**f, i**) in each cell were quantified. Data are presented as means ± SD of more than 300 cells from three biological replicates. Two-tailed unpaired Student's t-test. n.s., not significant. P values based on the order of appearance: in **e** (5.1E-39, 0.1121, 1E-8, 0.1259, 0.7436), in **f** (8.6E-51, 0.3748, 7.1E-27, 0.2346, 0.4682), in **h** (2.5E-12, 0.3563, 1.3E-5, 0.119, 0.2067) and in **i** (6.5E-46, 0.5748, 0.0007, 0.8471, 0.3907). Source data are provided in this paper or Mendeley database (https://data.mendeley.com/datasets/n9txt6y5cj/1).

References cited in Supplementary Information

1. Consortium, E.P. An integrated encyclopedia of DNA elements in the human genome. *Nature* **489**, 57-74 (2012).
2. Spruijt, C.G. *et al.* ZMYND8 Co-localizes with NuRD on Target Genes and Regulates Poly(ADP-Ribose)-Dependent Recruitment of GATAD2A/NuRD to Sites of DNA Damage. *Cell Rep* **17**, 783-798 (2016).
3. Davis, C.A. *et al.* The Encyclopedia of DNA elements (ENCODE): data portal update. *Nucleic Acids Res* **46**, D794-D801 (2018).
4. Chng, K.R. *et al.* A transcriptional repressor co-regulatory network governing androgen response in prostate cancers. *EMBO J* **31**, 2810-2823 (2012).
