# Supplementary material for: TRABID overexpression enables synthetic lethality to PARP inhibitor via prolonging 53BP1 retention at double-strand breaks
Source: Nat Commun. 2023 Mar 31;14:1810. doi: 10.1038/s41467-023-37499-5 (PMC10066190; doi:10.1038/s41467-023-37499-5)
Supplement: Supplementary file 3 — Reporting Summary [file 41467_2023_37499_MOESM3_ESM.pdf]

nature portfolio

Corresponding author(s): Haojie Huang

Last updated by author(s): Feb 26, 2023

Reporting Summary

Nature Portfolio wishes to improve the reproducibility of the work that we publish. This form provides structure for consistency and transparency in reporting. For further information on Nature Portfolio policies, see our Editorial Policies and the Editorial Policy Checklist.

Statistics

For all statistical analyses, confirm that the following items are present in the figure legend, table legend, main text, or Methods section.

n/a	Confirmed	
☐	☒	The exact sample size (n) for each experimental group/condition, given as a discrete number and unit of measurement
☐	☒	A statement on whether measurements were taken from distinct samples or whether the same sample was measured repeatedly
☐	☒	The statistical test(s) used AND whether they are one- or two-sided<br>*Only common tests should be described solely by name; describe more complex techniques in the Methods section.*
☐	☒	A description of all covariates tested
☐	☒	A description of any assumptions or corrections, such as tests of normality and adjustment for multiple comparisons
☐	☒	A full description of the statistical parameters including central tendency (e.g. means) or other basic estimates (e.g. regression coefficient) AND variation (e.g. standard deviation) or associated estimates of uncertainty (e.g. confidence intervals)
☐	☒	For null hypothesis testing, the test statistic (e.g. F, t, r) with confidence intervals, effect sizes, degrees of freedom and P value noted<br>*Give P values as exact values whenever suitable.*
☒	☐	For Bayesian analysis, information on the choice of priors and Markov chain Monte Carlo settings
☐	☒	For hierarchical and complex designs, identification of the appropriate level for tests and full reporting of outcomes
☒	☐	Estimates of effect sizes (e.g. Cohen's d, Pearson's r), indicating how they were calculated

Our web collection on statistics for biologists contains articles on many of the points above.

Software and code

Policy information about availability of computer code

Data collection	Bio-Rad CFX manager 3.1 was used to perform quantitative PCR. Leica Application Suit V4 was used to take IHC pictures. Carl Zeiss ZEN2 was used to take fluorescent pictures. Microsoft Excel version 2010 was used to process the data.
Data analysis	Graphing and statistical analysis: GraphPad Prism 8.0; DNA damage foci were quantified using the Image-Pro software(version 10). For each cell line analyzed with Image-Pro (version 10), 10 randomly picked photographs that included more than 200 cells were used to standardize foci counting and integration of optical density. The macs2 software were used for callpeak of CUT&Tag assay.

For manuscripts utilizing custom algorithms or software that are central to the research but not yet described in published literature, software must be made available to editors and reviewers. We strongly encourage code deposition in a community repository (e.g. GitHub). See the Nature Portfolio guidelines for submitting code & software for further information.

Data

Policy information about availability of data

All manuscripts must include a data availability statement. This statement should provide the following information, where applicable:

- Accession codes, unique identifiers, or web links for publicly available datasets
- A description of any restrictions on data availability
- For clinical datasets or third party data, please ensure that the statement adheres to our policy

The raw data for western blot generated in this study have been deposited in the Mendeley database (https://data.mendeley.com/datasets/n9txt6y5cj/1). The raw sequencing data for CUT&Tag have been deposited to the National Center for Biotechnology Information (NCBI) Gene Expression Omnibus (GEO) database with the

accession code GSE222267 (https://www.ncbi.nlm.nih.gov/geo/query/acc.cgi?acc=GSE222267). Source data are provided with this paper.

Human research participants

Policy information about studies involving human research participants and Sex and Gender in Research.

Reporting on sex and gender	The prostate cancer (male-specific cancer type) tissue microarray (TMA) slides were generated from only male patients, because the study investigated the pathology of prostate cancer.
Population characteristics	Archive tumor tissue collected at the First Affiliated Hospital of Xi'an Jiaotong University (Xi'an, China). 30 cases (age from 50-80) were selected solely based on their histopathology diagnosis and tumor tissue availability.
Recruitment	Samples were collected retrospectively from the First Affiliated Hospital of Xi'an Jiaotong University and annotated for major clinicopathologic variables through review of pathology reports and clinical records by trained personnel. Archive tumor tissue were selected solely based on their histopathology diagnosis and tumor tissue availability, and no other biases were present.
Ethics oversight	The studies were approved by the Ethics Committee of the First Affiliated Hospital of Xi'an Jiaotong University (Xi'an, China).

Note that full information on the approval of the study protocol must also be provided in the manuscript.

Field-specific reporting

Please select the one below that is the best fit for your research. If you are not sure, read the appropriate sections before making your selection.

☒ Life sciences ☐ Behavioural & social sciences ☐ Ecological, evolutionary & environmental sciences

For a reference copy of the document with all sections, see nature.com/documents/nr-reporting-summary-flat.pdf

Life sciences study design

All studies must disclose on these points even when the disclosure is negative.

Sample size	No sample size calculation was performed. Sample size was determined from similar experiments in the literature and for all experiments a minimum of three technical replicates were analyzed per sample.
Data exclusions	No data were excluded from the analyses.
Replication	Data are presented as the mean ± SD. Replicates of experiments are specified in figure legends
Randomization	In mouse experiments, all samples and animals are randomly divided into different experimental groups as indicated. For other studies, experiments were performed in large cell line populations, and randomization was therefore not appropriate, but the treated groups were attributed randomly.
Blinding	For mouse study, investigators were blinded for group allocation during data collection and analysis. For other studies, experiments were performed blinded.

Reporting for specific materials, systems and methods

We require information from authors about some types of materials, experimental systems and methods used in many studies. Here, indicate whether each material, system or method listed is relevant to your study. If you are not sure if a list item applies to your research, read the appropriate section before selecting a response.

Materials & experimental systems

n/a	Involved in the study	
☐	☒	Antibodies
☐	☒	Eukaryotic cell lines
☒	☐	Palaeontology and archaeology
☐	☒	Animals and other organisms
☒	☐	Clinical data
☒	☐	Dual use research of concern

Methods

n/a	Involved in the study	
☐	☒	ChIP-seq
☒	☐	Flow cytometry
☒	☐	MRI-based neuroimaging

Antibodies

Antibodies used	Primary antibodies used were TRABID (Abcam, # ab262879, 1:1000), 53BP1 (Abcam, # ab36823, 1:1000), HA.11 (Covance, #MMS-101R, 1:1000), SPOP (Proteintech Group, # 16750-1-AP, 1:1000), BRCA1 (Santa Cruz, # sc-642, 1: 500), RAD51 (Santa Cruz, # sc-377467, 1: 500), Myc (Santa Cruz, # sc-40, 1:1000), Flag (Sigma, # F-3165, 1:1000), ERK2 (Santa Cruz, # sc-1647, 1:1000), Phospho Histone H2A.X (S139) (Cell Signaling, # 9718, 1:1000), Phospho Histone H2A.X (S139) (Cell Signaling, # 80312S, 1:1000), LSD1 (Abcam, # ab129195, 1:1000), MBD3 (Abcam, # ab157464, 1:1000), Second antibodies were Rabbit IgG (H+L) Alexa Fluor 594 (Thermo Fisher, # A11037, 1:500), Rabbit IgG (Jackson ImmunoResearch, # 211-032-171, 1:5000), Mouse IgG (Jackson ImmunoResearch, #115-035-174, 1:5000), Mouse IgG (H+L) Alexa Fluor 488 (Thermo Fisher, # A11029, 1:500), Rat IgG (H+L) Alexa Fluor 488 (Life Technologies, # A-11006, 1:500), Mouse-IgGκ BP-FITC (Santa Cruz, # sc-516140, 1:500). Goat monoclonal (Millipore, #AP132, 1:100)
Validation	TRABID (Abcam, # ab262879, 1:1000), https://www.abcam.com/zranb1-antibody-ab262879.html;<br>53BP1 (Abcam, # ab36823, 1:1000), https://www.abcam.com/53bp1-antibody-ab36823.html;<br>HA.11 (Covance, #MMS-101R, 1:1000), https://www.biolegend.com/en-us/products/anti-ha-11-epitope-tag-antibody-11071?GroupID=GROUP26;<br>SPOP (Proteintech Group, # 16750-1-AP, 1:1000), https://www.ptglab.com/products/SPOP-Antibody-16750-1-AP.htm;<br>BRCA1 (Santa Cruz, # sc-642, 1: 500), https://www.scbt.com/p/brca1-antibody-c-20;<br>RAD51 (Santa Cruz, # sc-377467, 1: 500), https://datasheets.scbt.com/sc-377467.pdf;<br>Myc (Santa Cruz, # sc-40, 1:1000), Flag (Sigma, # F-3165, 1:1000), https://www.scbt.com/p/c-myc-antibody-9e10;<br>ERK2 (Santa Cruz, # sc-1647, 1:1000), https://www.scbt.com/p/erk-2-antibody-d-2;<br>Phospho Histone H2A.X (S139) (Cell Signaling, # 9718, 1:1000), https://www.cellsignal.com/products/primary-antibodies/phospho-histone-h2a-x-ser139-20e3-rabbit-mab/9718<br>Phospho Histone H2A.X (S139) (Cell Signaling, # 80312S, 1:1000), https://www.cellsignal.com/products/primary-antibodies/phospho-histone-h2a-x-ser139-d7t2v-mouse-mab/80312;<br>LSD1 (Abcam, # ab129195, 1:1000), https://www.abcam.com/kdm1lsd1-antibody-epr6825-nuclear-marker-and-chip-grade-ab129195.html;<br>MBD3 (Abcam, # ab157464, 1:1000), https://www.abcam.com/mbd3-antibody-epr9913-chip-grade-ab157464.html;<br>All of the antibodies used in this study were validated for the use in human specimens by the manufacturers and for the respective methods used in this manuscript (see home pages of respective manufacturers using catalogue numbers provided above).

Eukaryotic cell lines

Policy information about cell lines and Sex and Gender in Research

Cell line source(s)	The immortalized human embryonic kidney cell line 293T and prostate cancer cell lines PC-3 and human osteosarcoma cell line U2OS were purchased from ATCC (Manassas, VA).
Authentication	The cell lines were authenticated periodically via STR profiling (IDEXX BioResearch).
Mycoplasma contamination	All cell lines were tested negative of mycoplasma contamination.
Commonly misidentified lines (See ICLAC register)	No

Animals and other research organisms

Policy information about studies involving animals; ARRIVE guidelines recommended for reporting animal research, and Sex and Gender in Research

Laboratory animals	6-week-old SCID male mice were used for prostate cancer (male-specific cancer type) xenograft study as described in the Methods section. All mice were housed under standard conditions at room temperature with a 12 h light/dark cycle with access to food and water ad libitum and maintained under pathogen-free conditions. The animal study was approved by the Institutional Animal Care and Use Committee (IACUC) at the Mayo Clinic.
Wild animals	No
Reporting on sex	Only male mice were used in this study, because the study investigated the potential therapeutic use of PARP inhibitors in prostate cancer (male specific cancer type).
Field-collected samples	No
Ethics oversight	The animal study was approved by the Institutional Animal Care and Use Committee (IACUC) at the Mayo Clinic.

Note that full information on the approval of the study protocol must also be provided in the manuscript.

ChIP-seq

Data deposition

☒ Confirm that both raw and final processed data have been deposited in a public database such as GEO.

☒ Confirm that you have deposited or provided access to graph files (e.g. BED files) for the called peaks.

Data access links<br>*May remain private before publication.*	https://www.ncbi.nlm.nih.gov/geo/query/acc.cgi?acc=GSE222267
Files in database submission	NC_LSD1_R1.bw NC_LSD1_R1_IgG.bw NC_LSD1_R1_R1.fq.gz NC_LSD1_R1_R2.fq.gz NC_LSD1_R1_IgG_R1.fq.gz NC_LSD1_R1_IgG_R2.fq.gz<br>NC_LSD1_R2.bw NC_LSD1_R2_IgG.bw NC_LSD1_R2_R1.fq.gz NC_LSD1_R2_R2.fq.gz NC_LSD1_R2_IgG_R1.fq.gz NC_LSD1_R2_IgG_R2.fq.gz<br>IR_LSD1_R1.bw IR_LSD1_R1_IgG.bw IR_LSD1_R1_1.fq.gz IR_LSD1_R1_2.fq.gz IR_LSD1_R1_IgG_R1.fq.gz IR_LSD1_R1_IgG_R2.fq.gz<br>IR_LSD1_R2.bw IR_LSD1_R2_IgG.bw IR_LSD1_R2_1.fq.gz IR_LSD1_R2_2.fq.gz IR_LSD1_R2_IgG_R1.fq.gz IR_LSD1_R2_IgG_R2.fq.gz<br>NC_MBD3_R1.bw NC_MBD3_R1_IgG.bw NC_MBD3_R1_R1.fq.gz NC_MBD3_R1_R2.fq.gz NC_MBD3_R1_IgG_R1.fq.gz NC_MBD3_R1_IgG_R2.fq.gz<br>NC_MBD3_R2.bw NC_MBD3_R2_IgG.bw NC_MBD3_R2_R1.fq.gz NC_MBD3_R2_R2.fq.gz NC_MBD3_R2_IgG_R1.fq.gz NC_MBD3_R2_IgG_R2.fq.gz<br>IR_MBD3_R1.bw IR_MBD3_R1_IgG.bw IR_MBD3_R1_1.fq.gz IR_MBD3_R1_2.fq.gz IR_MBD3_R1_IgG_R1.fq.gz IR_MBD3_R1_IgG_R2.fq.gz<br>IR_MBD3_R2.bw IR_MBD3_R2_IgG.bw IR_MBD3_R2_1.fq.gz IR_MBD3_R2_2.fq.gz IR_MBD3_R2_IgG_R1.fq.gz IR_MBD3_R2_IgG_R2.fq.gz
Genome browser session<br>(e.g. UCSC)	https://www.ncbi.nlm.nih.gov/geo/query/acc.cgi?acc=GSE222267

Methodology

Replicates	Each sample were replicated twice
Sequencing depth	The total sequencing depth for raw data of each sample ranged from 6 to 60 million, the Unique Mapped reads ranged from 6 to 56 million, the fragment sizes for read pairs were calculated by a BAM file from paired-end sequencing (2 x 50 bp minimum).
Antibodies	LSD1 (Abcam, # ab129195, 1:1000), MBD3 (Abcam, # ab157464, 1:1000)
Peak calling parameters	The bam file generated by the unique mapped reads as an input file, using macs2 software for callpeak with cutoff qvalue < 0.05.
Data quality	Raw data (raw reads) of fastq format were firstly processed through in-house perl scripts. In this step, clean data (clean reads) were obtained by removing reads containing adapter, reads containing ploy-N and low quality reads from raw data. At the same time, Q20, Q30 and GC content the clean data were calculated. All the downstream analyses were based on the clean data with high quality.
Software	The size distribution of libraries was determined by Agilent 4200 TapeStation analysis, and libraries were mixed to achieve equal representation as desired aiming for a final concentration as recommended by the manufacturer. Sequencing was performed in the Illumina Novaseq 6000 using 150bp paired-end following the manufacturer's instructions. The bam file generated by the unique mapped reads as an input file, using macs2 software for callpeak with cutoff qvalue < 0.05.
